# Computing DNA duplex instability profiles efficiently with a two-state model: trends of promoters and binding sites

**DOI:** 10.1186/1471-2105-11-604

**Published:** 2010-12-21

**Authors:** Miriam R Kantorovitz, Zoi Rapti, Vladimir Gelev, Anny Usheva

**Affiliations:** 1 Department of Mathematics, University of Illinois at Urbana-Champaign, Urbana, IL, USA; 2 National Center for Supercomputing Applications, University of Illinois at Urbana-Champaign, Urbana, IL, USA; 3 Beth Israel Deaconess Medical Center, Harvard Medical School, Boston, MA, USA

## Abstract

**Background:**

DNA instability profiles have been used recently for predicting the transcriptional start site and the location of core promoters, and to gain insight into promoter action. It was also shown that the use of these profiles can significantly improve the performance of motif finding programs.

**Results:**

In this work we introduce a new method for computing DNA instability profiles. The model that we use is a modified Ising-type model and it is implemented via statistical mechanics. Our linear time algorithm computes the profile of a 10,000 base-pair long sequence in less than one second. The method we use also allows the computation of the probability that several consecutive bases are unpaired simultaneously. This is a feature that is not available in other linear-time algorithms. We use the model to compare the thermodynamic trends of promoter sequences of several genomes. In addition, we report results that associate the location of local extrema in the instability profiles with the presence of core promoter elements at these locations and with the location of the transcription start sites (TSS). We also analyzed the instability scores of binding sites of several human core promoter elements. We show that the instability scores of functional binding sites of a given core promoter element are significantly different than the scores of sites with the same motif occurring outside the functional range (relative to the TSS).

**Conclusions:**

The time efficiency of the algorithm and its genome-wide applications makes this work of broad interest to scientists interested in transcriptional regulation, motif discovery, and comparative genomics.

## Background

DNA duplex instability is manifested as the ease at denaturating the DNA double strand, i.e., as the partial melting and unfolding of double stranded DNA. The study of DNA duplex instability has been a fascinating subject for many reasons: its importance for techniques such as PCR, sequencing by hybridization, antigene targeting, and for understanding replication, mutation, repair, and transcription, see [1] and references therein.

With respect to understanding transcription initiation, in the very recent past, there has been increased evidence that duplex instability, as well as other physiochemical properties, reveal specific signatures of TSS and core promoter elements. In this context, there have been several comprehensive analyses of genomes such as that of the Plasmodium falciparum [2], yeast [3], human, and other animals [4-7]. To a smaller scale, structural properties of DNA have also been used to predict DNA function in viral sequences [8,9].

It has also been shown that the DNA duplex instability profiles can be used to aid motif discovery in yeast [3]. The instability profiles, computed with the on-line tool WebSIDD [10], were used to derive informative positional priors and incorporated into a motif finding algorithm. As a result, the performance of the motif finding program improved significantly.

The need for an efficient method to compute the profiles was stressed in [3], since the on-line tool WebSIDD could not be used to efficiently compute profiles of sequences that were several thousands base pairs long. The algorithms used for computing DNA instability profiles for the above applications [2-9] either have non-linear time complexity (such as the algorithms based on the Peyrard-Bishop-Dauxois (PBD) model [11,12] and WebSIDD, based on the Benham model [13,14]) or are linear time approximations to a non-linear-time model (e.g., [6]).

Some progress in this direction has been made. Recently, in [15] the Zimm-Brag model was used for a genome wide comparison between coding domains and thermodynamically stable regions. In some organisms the corellation between coding domains and thermodynamic stability allowed the identification of putative exons or genes. The authors state that the algorithm is linear in the length of the sequence. Also, using the Poland-Scheraga model in [16] another algorithm for DNA melting calculations was reported with time complexity less than quadratic.

In this work we modified an Ising-type model [17-19] that identifies as major contributions to DNA stability the hydrogen bonds between the complementary bases and the nearest-neighbor stacking interaction. One of the advantages of this model is that it can be implemented efficiently, since the time complexity of the algorithm is linear in the length of the sequence.

Another feature of the method we use is that it directly computes the probability of bubble formation of any size *k*. Our operational definition of a bubble is that of a strand separation, or DNA 'opening' spanning several base pairs. Here, a bubble of size *k *means that at least *k *base pairs are open.

Studies have suggested that the ability of the DNA to form a 'transcriptional bubble' at the transcriptional start site is essential to initiate transcription. Using the PBD model, in [20], it was argued that thermodynamic instability profiles are able to identify the location of TSS. In [21] it was demonstrated that bubble size is important, in the sense that when the simulated bubble size equals the transcriptional bubble size, the highest peak in the instability profile appears at the TSS. Most previous algorithms (in particular, [3,4,6]), only compute the opening probabilities of one base-pair at a time and use averaging techniques to measure the propensity of the DNA to form a bubble of size *k >*1 at a specific location. This averaging process is not equivalent to computing the opening probability of the whole window of size *k*.

Using the Ising-type model, we computed the DNA instability profiles for the human promoter regions in Database of Transcriptional Start Sites (DBTSS). We show that these profiles provide an insight into core promoter elements such as the downstream promoter element (DPE), transcription factor II recognition element (BRE), initiator (Inr) and GC box. We show that there is an association between the location of local extrema in the instability profiles and the presence of core promoter elements at these locations. We present evidence that BRE and DPE prefer stability, whereas the TATA box and the Inr prefer instability.

Finally, we examine the applications of the DNA duplex instability profiles to motif discovery. Our findings raise a concern that the "one size fits all" approach to transcription factors used in [3], may not be appropriate.

### Related approaches

Most of the approaches for the computation of DNA instability profiles use models that are coarse-grained, in the sense that they take into account only the major contributions to DNA stability.

The Peyrard-Bishop-Dauxois model [11,12] assumes that the hydrogen bonds and stacking interaction are the main contributions to DNA stability. Like the modified Ising-type model, it does not take into account explicitly the three-dimensional structure of the double helix and neglects torsional effects. The main difference with the approach we use in this work is that the variable that describes the stretching of the bonds is continuous rather than discrete. The computational complexity of this model is non-linear in the length of the sequence. By direct integration an algorithm was devised in [22] that reduces the complexity of function evaluations to being linear in the length of the sequence and quadratic in the number of grid points used in the integration.

The Benham model [10,13,14] uses the free energy needed to separate the two strands and destroy the helical structure as a measure of instability. This model predicts the location and extend of destabilization given the DNA sequence and imposed super-helical stress and is discrete: the base pair is assumed to be either separated or not. The Benham model has non-linear time complexity.

In [6] the human genomic melting map was obtained based on the Poland model [23], which uses recurrence relations to calculate the probabilities of transition of the double helix from the helical to the coil state, rather than considering the state of the hydrogen bonds and stacking interactions. The approach we use here is more general since it allows for the study of localized openings that are precursors to melting, instead of considering only the complete melting of a DNA sequence. The algorithm used in [6] is a linear time approximation to the non-linear-time Poland model.

In [4] an approach to predict promoters in whole-genome sequences was used, with the aid of large-scale structural properties of DNA, such as GC content, stabilizing energy of Z-DNA, DNA denaturation values, protein induced deformability, and duplex free energy. First, structural profiles are calculated by converting the nucleotide sequence into a numerical profile, by replacing each di-or trinucleotide with its corresponding structural value. Next, the values are averaged over a window of size 400. The approach we use is different in the sense that no averaging is taking place, rather we calculate conditional probabilities of having *k *base pairs in the open state and loss of stacking interactions. The cooperative and long range effects in the Ising type model used here, are due to that fact that in the calculation of the probabilities, the entire sequence is taken into account in the evaluation of the partition function (normalizing constant so the probabilities at a given base pair add up to one). In this sense, our approach is an effective smoothing and no averaging within the same sequence takes place.

Other approaches to the study of DNA denaturation include the examination of the breathing dynamics from a probabilistic point of view [24] and [25]. In [24] the authors develop a master equation, which together with a Gillepsie algorithm, generates sequence-specific stochastic time series of partially melted regions in DNA. In [25] the dynamics and thermodynamics of twist-induced denaturation was studied in a long, random sequence of DNA, using large deviation theory, scaling arguments, and Monte Carlo simulations.

## Results and Discussion

### The model

Our results are based on the calculation of the thermal equilibrium statistical properties of dsDNA using a modified version of the model introduced in [17]. The model was proposed as a tool to study the thermal fluctuations that lead to the infrequent events of the Watson-Crick base-pair opening, also referred to as DNA breathing. This fluctuational base-pair opening implies the disruption of hydrogen bonds between the complementary bases and the loss of stacking interactions between adjacent base-pairs by the flipping of the base pair out of the helical stack.

Like other models that are designed to predict the propensity of DNA to breathe (such as [11]), this model takes into account two major contributions to DNA stability: the lateral pairing between the complementary bases and the stacking interactions of the pairs with both immediate neighbors along the helical axis. The model in [17] also introduced a novel term accounting for the unfavorable positioning of the exposed base, which proceeds through the formation of a highly constrained small loop, and was described as the ring factor. In this work, we neglect the ring factor, since quantitatively it was found to be an adjustable parameter and in our simulations it had the effect of mainly translating vertically the opening propensity profiles - the plot of the propensity to open of base-pair *n *vs. *n*.

This Ising-type model distinguishes two states of base-pairs, the *open *state in which the hydrogen bonds are broken and the bases are flipped out of the stack, and the *closed *state in which the opposite is true. The instability profiles are obtained by calculating the probability *P_k_*(*n*) for *k *consecutive base-pairs to be open at the same time, starting at base pair *n*. The parameter *k *is called the bubble-size. In the original version of the model [17] only the case *k *= 1 was considered. In this work, we generalized the model to be able to calculate the propensity *P_k_*(*n*) for *k ≥ *1. Our choice for *k *in this work varies from *k *= 1 to *k *= 9. The need to consider more than one value of *k*, stems from the fact that new features of the opening profiles emerge with different values. For example, *k *= 1 gives an implementation of the original model introduced in [17], but it tends to be noisy (see examples in [Additional file 1]).

It is important to note that the way in which the opening probabilities are calculated by our method for a bubble of size *k >*1 is fundamentally different from considering the probabilities that *each individual base-pair is open *and then averaging over a window of size *k*. Our method computes the probability that *all k *base-pairs in the window of size *k *are open *simultaneously*.

The inhomogeneity of the sequence is taken care of by 2 sets of parameters for the hydrogen bonds and 10 parameters for the stacking interaction of the adjacent bases. There are no free parameters in this approach. The thermodynamic parameter values used in our simulations are the ones reported in [18,19] and were determined experimentally [18].

The approach we use provides an efficient method for a genome-wide scan: the time complexity of our algorithm is linear in the length of the sequence. It took less than one second to compute the profile of a 10,000 bp long sequence, while it took more than two hours for WebSIDD [10].

### Average Profiles of human, mouse and zebrafish promoter sequences

**Figure 1:**
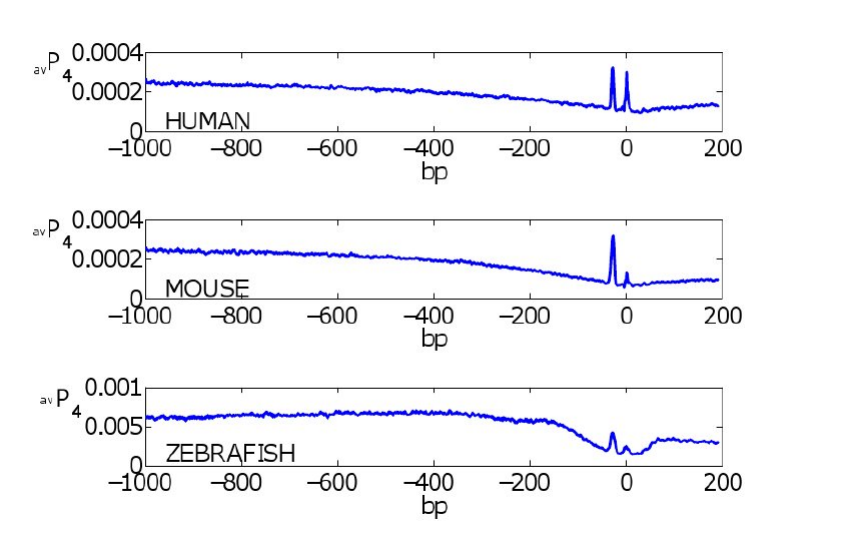
**Average profiles of human, mouse, and zebrafish promoter sequences**. The averaged opening profile of the promoter sequences in human, mouse, and zebrafish. The bubble size is 4.

The average profile of the promoter sequences from Database of Transcriptional Start Sites (DBTSS) is shown in Figure 1 (k = 4). There are two clear peaks at about -30 and 1 relative to the TSS. The peaks coincide with the location of the TATA box and the Inr/TSS, respectively. We also see a trend of the opening probabilities to decrease toward the TSS and increase after the TSS. This is similar to the trend observed in other DNA physical-properties profiles, see for example [5] or [4].

### Signatures of the human core promoter elements

**Figure 2:**
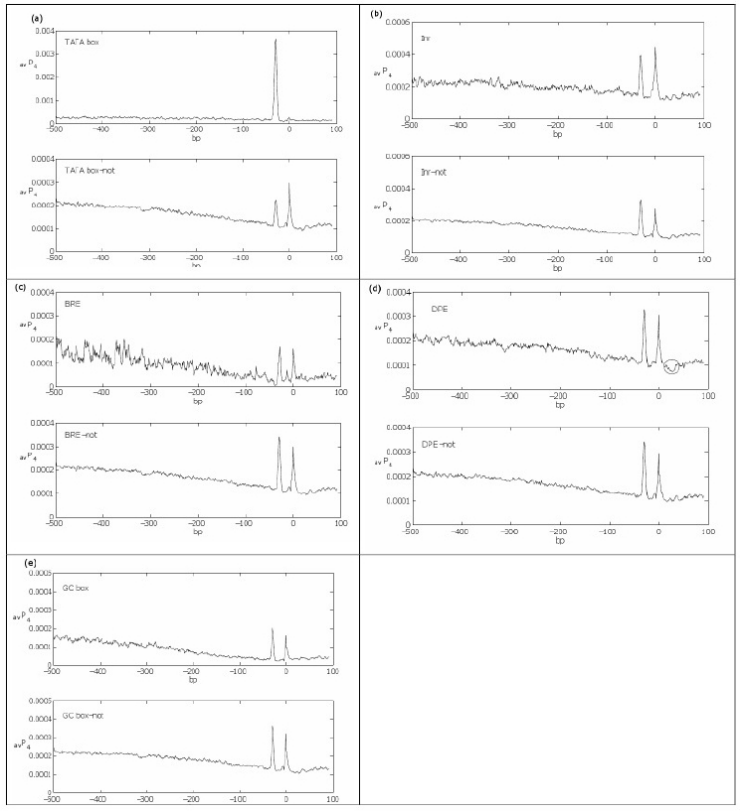
**Signature of the human core promoter elements**. We show the average profiles of all sequences that are (a) containing and lacking the TATA box, (b) containing and lacking Inr, (c) containing and lacking BRE, (d) containing and lacking DPE, and (e) containing and lacking the GC box. The bubble size is 4. In (d), the DPE's signature is circled.

**Figure 3:**
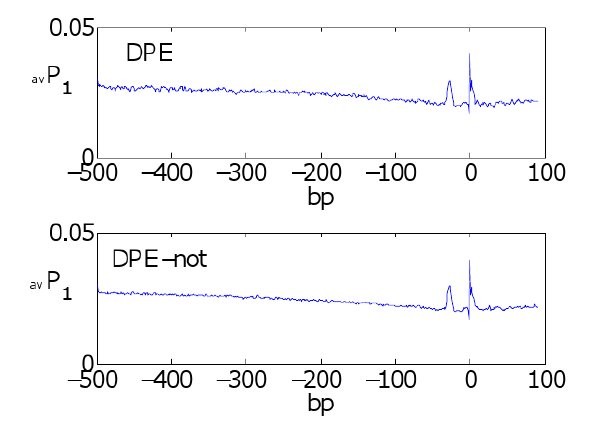
**Bubble size effects signature**. The averaged profile using bubble size *k *= 1 of the sequences containing and lacking the DPE motif did not produce an apparent signature for DPE.

To obtain a signature for a given core promoter element, we compared the average profile of all sequences classified as containing the functional promoter element vs. the average profile of the complement set of sequences (see methods section). The average profiles are shown in Figure 2 (*k *= 4). The TATA box has a clear signature of a high peak about -30. The Inr's signature seems to be a higher peak at the TSS together with a higher baseline around TSS. BRE's signature is a dip at about -40 together with a lower baseline on [-100, 100]. DPE's signature is a low dip around +25. Note that the *k *= 1 average profiles did not detect a signature for DPE, see Figure 3. The signature for the GC box is an overall lower baseline. The signatures of these core promoters suggest that the TATA box and Inr prefer DNA instability while BRE and DPE prefer DNA stability.

"Shape recognition" of DNA is a major determinant in protein-DNA interactions [26]. Examination of DNA-protein complex structures has revealed that transcription factor (TF) binding sites can exhibit characteristic structural signatures, e.g. in terms of deformability [27], bending, groove width, or the presence of kinked bases [26]. These properties may in some instances be correlated to thermodynamic stability and the presented here characteristic profiles for the various promoter elements may reflect conformational properties of the corresponding DNA-protein complexes. In the case of the TATA box, the relationship is easy to see. The TATA box binding protein (TBP) unwinds and bends the DNA double helix almost at 90 degree angle to achieve specific binding [28], suggesting that sequences that are resistant to such deformation would not bind TBP well. For the Inr element, it has been proposed that a propensity for strand separation assists in the formation of the "transcriptional bubble" [29], the exposed single strand DNA required by RNA polymerases to initiate transcription. Moreover, YY1 transcription factor, which recognizes Inr motifs such as CCATTT, makes specific contacts with one strand only [30,31], raising the possibility that its binding also assists in formation of the transcriptional bubble. Generally however, DNA conformational properties are determined by a complex interplay of hydrogen bonding, base stacking energies, hydration, counterions, and steric effects well past the predictive ability of a simple thermodynamic stability model.

### DNA instability scores differentiate functional binding sites from non-functional binding sites

For each motif, we compared the scores of its functional sites versus its non-functional sites. We classified an occurrence of a motif in a sequence as a functional site if it occured within the functional window for the motif, as specified in Table 1. Occurrences of motifs outside their functional window were classified as non-functional sites. For example, for the Inr motif YYANWYY, we divided the sites where YYANWYY occurred into two sets: functional sites and non-functional sites. The set of functional sites consisted of all sites, in all sequences considered, where YYANWYY occurred between -5 and +6 relative to the TSS. The set of non-functional sites consisted of all other sites where YYANWYY occurred. We then compared the distribution of the instability scores at the functional YYANWYY sites with the distribution of the scores of the non-functional YYANWYY sites.

**Table 1:** Core promoter elements, their consensus sequences and functional window

Name	Consensus Sequence	Functional Window
TATA box	TATAWA	-33 to -23

Inr	YYANWYY	-5 to +6

DPE	RGWYV	+23 to +33

BRE	SSRCGCC	-42 to -32

GC box	GGGCGGG	-170 to -5

Core promoter element motifs and functional windows, with the IUPAC convention of *R *= [*GA*], *Y = *[*TC*], *W *= [*AT*], *S *= [*GC*], *V *= [*GCA*] and *N *= [*AGCT*].

**Figure 4:**
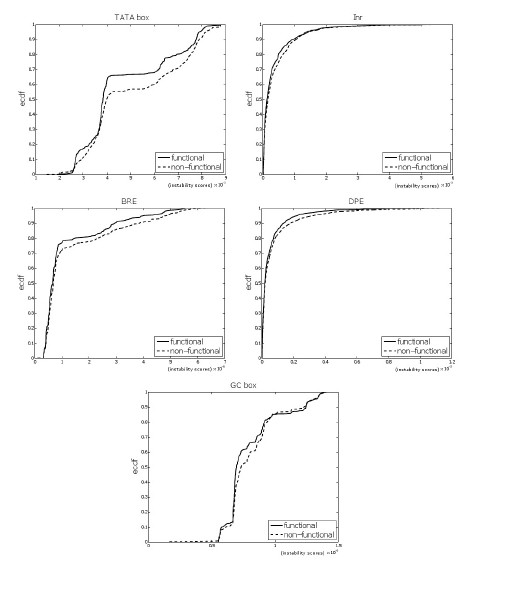
**Functional vs. non-functional TFBS**. The figure shows the empirical cumulative distribution functions of the functional and non-functional binding sites for the TATA box (p-value 6.4 10^-7^), Inr (*p *= 1.2 10^-5^), BRE (*p *= 0.01758), DPE (*p *= 3.8 10^-6^) and GC box (*p <*2.2 10^-16^). The black lines correspond to the functional binding sites, while the broken lines correspond to the on-functional sites. The bubble size is *k *= 4.

For each motif, we found that the distribution of the scores of the functional sites was significantly different than the distribution of the scores of the non-functional sites (see Figure 4). We observed that, per motif, the scores of the functional sites were *lower*, on average, than the scores of non-functional sites. Figure 4 shows that per motif, the graph of the empirical cumulative distribution function (ecdf) of the functional sites lies *above *the ecdf of the non-functional sites. It means that, regardless of the GC content of the motif, the scores of the functional binding sites were *lower *in general than those of the non-functional binding sites. This result suggests that, per motif, functional binding site prefer stability when compared with non-functional binding sites.

### DNA instability scores of functional binding sites vs. random sites

**Figure 5:**
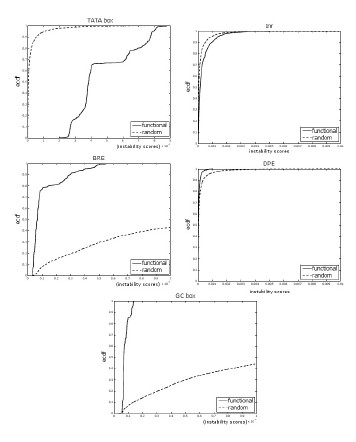
**Functional TFBS vs. random sites**. The empirical cumulative distribution functions of functional binding sites vs. random sites for the TATA box, Inr, BRE, DPE, and GC box. All with p-value *<*2.2 10^-16^. The bubble size is *k *= 4.

For each core promoter motif, we compared the distribution of the DNA instability scores of the functional sites with the distribution of the scores of random sites. The random sites were picked at random from the same promoter sequences that the functional motif sites where found (see methods section). Random sites, in general, do not share any common feature. For each motif, the distribution of the scores of the functional sites was significantly different than the distribution of the scores of random sites (see Figure 5).

We observed that for the TATA box and Inr, the scores of the functional sites were *higher*, on average, than the scores of random sites. On the other hand, for BRE, DPE and GC box, the scores of the functional sites were *lower*, on average, than the scores of random sites. Figure 5 shows that for TATA box and Inr, the graph of the ecdf of the functional sites lies *below *the ecdf of the random sites, while for BRE, DPE and GC box, the ecdf of the functional sites lies *above *the ecdf of the random sites. This suggests that functional sites of TATA box and Inr "prefer" *less *stability but BRE, DPE and GC box "prefer" *more *stability, when compared to *random *sites.

In [3], Gordân et al. incorporated the DNA instability profiles into a motif finding algorithm based on the following observation regarding high-confidence transcription factors binding sites ("functional TFBS") in yeast. They noticed that the the distributions of the instability scores were significantly different for the high-confidence TFBS compared to *random *sites. This information was then used to derive informative positional priors.

Gordân et al. also observed that, when their set of high-confidence yeast TFBS was compared with *random *sites, it had, in general, lower instability scores. They hypothesized that TFBS occur preferentially in regions with high DNA duplex *stability*.

Our findings for *individual *core promoter elements in human suggest that, compared to *random *sites, TFs with AT-rich motifs prefer *instability *while GC-rich motifs prefer stability. This is consistent with our results on the human core promoters signature. We hypothesize that the set of Yeast motifs used in [3] was GC-rich, therefore skewing the results when compared to random sites and improving the overall performance of the motif discovery tool on the GC-rich motif data set. This relationship between the GC content and stability preference is supported by the following results on shuffled motifs.

### DNA instability scores of functional binding sites vs. shuffled motif sites

In this context, a shuffled motif is a biologically meaningless motif created from the original core promoter motif by shuffling the regular expression for the motif. For example, for the Inr motif YYANWYY, a shuffled motif can be ANYYYYW. In this case we considered the instability scores at all sites in the sequences where ANYYYYW occurred.

**Figure 6:**
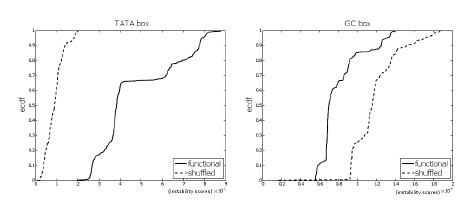
**Functional TFBS vs. shuffled motif sites**. The empirical cumulative distribution functions of functional binding sites vs. shuffled-motif sites for the TATA box (*p <*2.2 10^-16^) and GC box (*p <*2.2 10^-16^). The bubble size is *k *= 4.

For each core promoter motif, we compared the distribution of the DNA instability scores of shuffled-motif sites with the distribution of the scores of random sites. The results were similar to the previous comparison of *functional motif sites *with random sites. The two distributions were significantly different. For the shuffled motifs that were highly AT-rich (such as a shuffled TATA box), the graph of the ecdf of the shuffled motif sites lied *below *the ecdf of the random sites, while for the GC-rich shuffled-motifs (such as shuffled BRE and GC box) the ecdf of the functional sites lied *above *the ecdf of the random sites (see Figure 6).

These results show that, on average, the AT-rich shuffled motifs scored higher (more instability) than random sites, while the GC-rich shuffled motifs scored lower than random sites.

**Figure 7:**
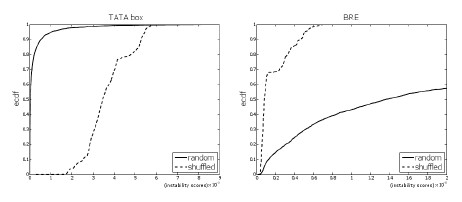
**Shuffled motif sites vs**. random sites. The empirical cumulative distribution functions of shuffled-motif sites vs. random sites for the TATA box (*p <*2.2 10^-16^) and BRE (*p <*2.2 10^-16^). The bubble size is *k *= 4.

We also compared the distribution of the instability scores of functional motif sites with the distribution of the scores of the shuffled-motif sites. For each core promoter motif, the two distributions were significantly different. For the highly AT-rich motifs, such as the TATA box, the instability scores of the functional binding sites of the motifs were, in general, higher than the scores of the shuffled motif sites. For the highly GC-rich motifs, such as the GC box, the scores of the functional sites were lower in general, than the scores of the shuffled motif sites (see Figure 7).

It is not surprising that (per motif) the two distributions compared were significantly different. The instability score of a word at a site depends in large on the content of the word. Therefore it is expected that scores at sites of one selected set of words and scores of sites of a different set of words (or random words) will have different distributions. But it is important to note how the GC content of the motif effect the results. These results suggest that binding sites for different TFs have different instability profiles when compared to random sites, even when the GC content of the random sites is similar to the GC content of the TFBS. Therefore, in order to capture biologically significant features, one should be careful when combining instability scores of binding sites from different TFs.

## Conclusions

We have introduced a linear time algorithm for computing DNA duplex instability profiles. The algorithm has the feature that it can compute the probability of formation of localized openings of any size *k*. Our analysis has shown that when studying the signatures of functional sites, bubble size matters. Specifically, considering the case of one base pair open, which corresponds to case *k *= 1, in some instances fails to identify the signatures. With our method, one can easily perform the calculation with several bubble sizes and be able to differentiate the signatures.

Our study has shown that core promoters with GC-rich motifs prefer stability, while those with AT-rich motif prefer instability. We have also shown that the DNA instability scores can differentiate functional binding sites from non-functional binding sites. We have demonstrated that a fast algorithm for the calculation of instability profiles can be a powerful tool in the investigation of entire genomes, with potential applications to motif discovery.

## Methods

### The model

A standard statistical mechanical approach to calculate the propensity of base-pair opening in dsDNA is applied. The total partition function for a sequence whose length is *N *base-pairs reads

$$Z=\sum_{\begin{array}{c} \sigma_{j}=0,1\\ j=1,...,N \end{array}}\prod_{i=1}^{N}{\left(\delta_{i}\right)}^{\sigma_{i}}{\left(\alpha_{i}\right)}^{\sigma_{i}}{\left(\delta_{i+1}\xi \right)}^{f\left(\sigma_{i},\sigma_{i+1}\right)},$$

where *δ*_1 _= 1, *δ*_*N*+1 _= 1 and *σ*_*N*+1 _= 1. Here, *σ*_i _= 0, 1 corresponds to the closed and open state of the base-pair, respectively in position *i*, and

$$f\left(\sigma_{i},\sigma_{i+1}\right)=\{\begin{array}{cc} \sigma_{i} & \text{if}\;\sigma_{i+1}=0\;\\ 0 & \text{if}\;\sigma_{i+1}=1,\; \end{array}$$

and *α_i _*and *δ_i _*are the base-pairing and base-stacking parameters, respectively. *ξ *is the ring factor (entropic factor parameter) introduced in [17]. The base-pairing and base-stacking parameters used in our simulations are given by

$$\alpha_{i}=\exp\left(\frac{\Delta G_{i}^{BP}}{RT}\right)\;\;\text{and}\;\;\delta_{i}=\exp\left(\frac{\Delta G_{i-1,i}^{ST}}{RT}\right)\;,$$

where *R *is the gas constant, *T *is the temperature, the values of $\Delta G_{i}^{BP},\;\Delta G_{i-1,i}^{ST}$ are shown in Table 2.

**Table 2:** Stacking and base-pairing parameters

$\Delta G_{KL}^{ST}$	KL	A	T	G	C
	A	-1.49	-1.72	-1.44	-2.19

	T	-0.57	-1.49	-0.93	-1.81

	G	-1.81	-2.19	-1.82	-2.55

	C	-0.93	-1.44	-1.29	-1.82

	AT		0.64		

*ΔG^BP^*	GC		0.12		

Hydrogen-bond and base-stacking parameter values used in the numerical calculations of the destabilization profiles.

The conditional partition function with *k *consecutive base-pairs open in positions *n*, *n *+ 1,..., *n *+ *k - *1 is given by

$$Z_{k}(n)=\sum_{\begin{array}{c} \sigma_{j}=0,1\\ j\in \left[1,N\right]\backslash \left[n,n+k-1\right] \end{array}}\prod_{i=1}^{N}g_{i},$$

where

$$g_{i}={\left(\delta_{i}\right)}^{\sigma_{i}}{\left(\alpha_{i}\right)}^{\sigma_{i}}{\left(\delta_{i+1}\xi \right)}^{f(\sigma_{i},\sigma_{i+1})},$$

and with *δ*_1 _= 1, *δ*_*N*+1 _= 1, *σ*_*N*+1 _= 1, and *σ_j _*= 1 for *j *= *n,..., n *+ *k *- 1. The opening propensity of base-pairs *n *through *n *+ *k *- 1 is the ratio of the two partition functions

$$P_{k}\left(n\right)=\frac{Z_{k}\left(n\right)}{Z}.$$

We used a MATLAB (The MathWorks, Natick, MA) program to calculate the partition functions in the equations above directly, using the matrix representation described in [17] that reduces the calculations of the partition functions to matrix multiplications. For example:

$$Z=\left(1\;\;1\right)\;\prod_{i=1}^{N-1}A_{i}\;\left(\begin{array}{c} 1\\ 0 \end{array}\right),$$

where

$$A_{i}=\left(\begin{array}{cc} 1 & 1\\ \delta_{i}\alpha_{i}\delta_{i+1}\xi & \delta_{i}\alpha_{i} \end{array}\right)\left(\begin{array}{cc} 1 & 1\\ \delta_{N}\alpha_{N} & \delta_{N}\alpha_{N} \end{array}\right).$$

For all the computations in this paper, the temperature parameter, *T *, was set to 37*C *and the ring factor parameter, *ξ*, to 1.

### Data

Promoter sequences were obtained from the DBTSS website, version 6 [32]. Only sequences with NM ids were considered, and redundancies were dealt with by choosing one representative at random. For human, the total number of sequences considered was 15,194, for mouse 15,337 and for zebrafish 5,343.

### Average Profiles

Given a collection *C *of *N *promoter sequences, the average score at position *n *relative to TSS is

$$avg_{k}\left(n\right)=1/N\sum_{S\in C}P_{k}\left(n_{S}\right),$$

where *n_S _*is position *n *(relative to TSS) in sequence *S *and *P*_k_(*n_S_*) is the opening propensity of *k *base pairs being open, starting at position *n_S_*.

### Signature of the human core promoter elements

Five core promoters were considered: the transcription factor II recognition element (BRE), the downstream promoter element (DPE), initiator (Inr), the TATA box, and the GC box. The sequence motifs and functional windows are shown in Table 1.

#### Sequence classification

A sequence was classified as containing a given core promoter if the motif for that core promoter had a match inside the appropriate functional window. A match here is an exact match of the regular expressions given in Table 1 on the positive strand.

### DNA instability scores differentiate functional binding sites from non-functional binding sites

For a given motif, the sites where the motif occurred in the sequences where divided into two sets: functional sites and non-functional sites. The functional sites were those inside the functional window. Non-functional sites were sites outside the functional window with a buffer zone of 10 base-pairs. The count of all non-overlapping sites is given in Table 3. Note that one can have more than one non-overlapping motif in a functional window.

**Table 3:** Number of functional sites and non-functional sites per motif

	functional sites	non-functional sites
TATA box	513	3,582

Inr	2,015	59,525

BRE	315	11,029

DPE	3,817	154,847

GC box	3,601	2,557

The count of functional and non-functional sites where each core promoter occurs.

For each motif site we assigned an average score as follows. For *k *smaller than the motif length, we took the average opening probabilities of the *k*-windows that are contained in the site. For *k *greater than the motif length, we averaged the scores of the *k*-windows that *contained *the site. The distributions of these scores of the functional sites was compared to the distribution of the scores of the non-functional sites using two sample Kolmogorov-Smirnov test.

### DNA instability scores of functional binding sites vs. random sites

For each functional site we picked at random 10 sites of equal length from the same promoter region. For each site we assigned an average score as was done for the functional sites.

### DNA instability scores of functional binding sites vs. shuffled motif sites

For each core promoter we tested several shuffles of its motif. Note that some motifs do not have many non-redundant shuffles. For example, the GC box motif, GGGCGGG, has only 7 non redundant shuffles. The tests were performed for one shuffle at a time. For each core promoter, the different shuffles produced similar results. The results shown in the Results Section are for one representative shuffle per core promoter (see Table 4).

**Table 4:** Shuffled motifs

	shuffled motif
TATA box	ATAWTA (3,252)

Inr	ANYYYYW (80,943)

BRE	RGCSCSC (12,297)

DPE	YRWVG (142,874)

GC box	GCGGGGG (2,618)

The shuffled motifs used for the figures and the number of non-overlapping sites for the shuffled motifs.

## Authors' contributions

MRK participated in the design of the study, performed the tests and statistical analysis and wrote parts of the manuscript. ZR designed and implemented the algorithm, participated in the design of the study and statistical analysis, and wrote parts of the manuscript. VG participated in the design of the study and wrote parts of the manuscript. AU conceived of the study, and participated in its design and helped to draft the manuscript. All authors read and approved the final manuscript.

## Supplementary Material

Additional file 1 **Examples of DNA duplex instability profiles**. This file contains figures showing the DNA duplex instability profiles for two promoter sequences, with bubble size ranging from k = 1 to k = 9 for each sequence. The genes used for these examples are CFTR and TJP2. Click here for file
