# Supplementary material for: Computing DNA duplex instability profiles efficiently with a two-state model: trends of promoters and binding sites
Source: BMC Bioinformatics. 2010 Dec 21;11:604. doi: 10.1186/1471-2105-11-604 (PMC3018474; doi:10.1186/1471-2105-11-604)

Supplementary Figures

Examples of DNA duplex instability profiles

The following figures show the DNA duplex instability profiles for two promoter sequences, with choice of the bubble size parameter ranging from $k = 1$ to $k = 9$ for each sequence. The genes used for these examples are:

- *Homo sapiens cystic fibrosis transmembrane conductance regulator (CFTR)*, RefSeq ID: *NM_000492*.
- *Homo sapiens tight junction protein 2 (zona occludens 2) (TJP2)*, RefSeq ID: *NM_004817.*

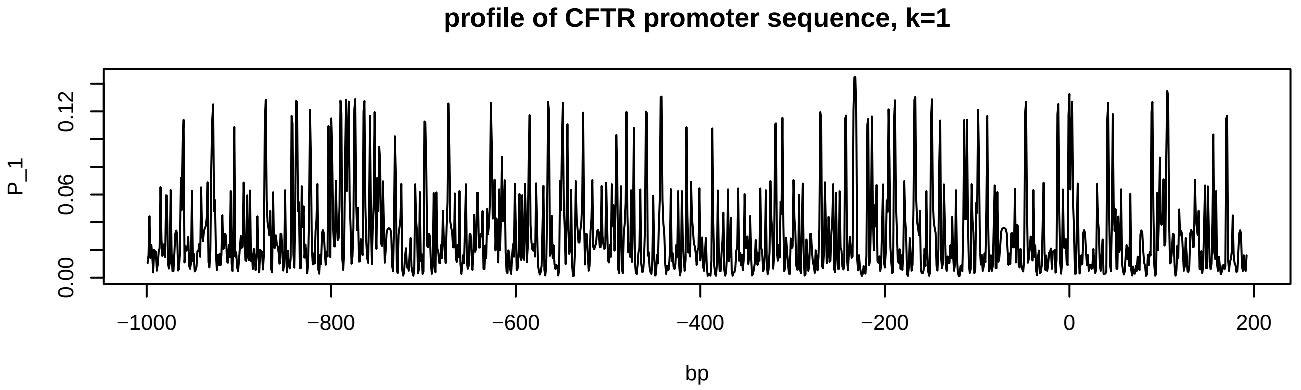
profile of CFTR promoter sequence, k=1
P_1
0.00
0.06
0.12
−1000
−800
−600
−400
−200
0
200
bp

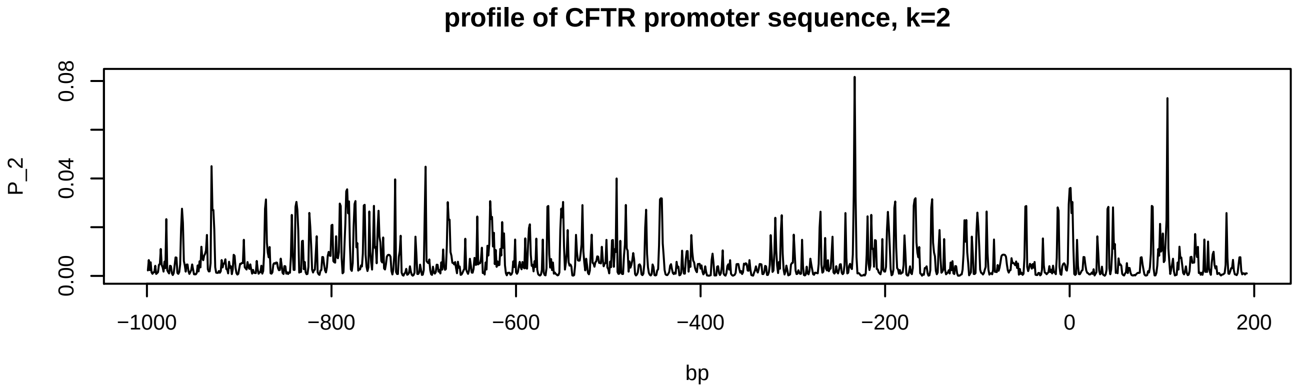
profile of CFTR promoter sequence, k=2
P_2
0.00
0.04
0.08
−1000
−800
−600
−400
−200
0
200
bp

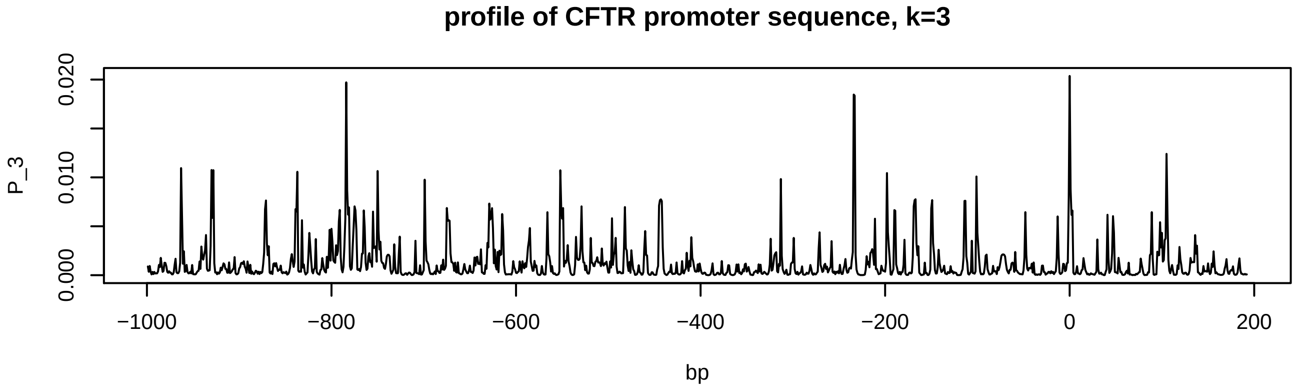
profile of CFTR promoter sequence, k=3
P_3
0.000
0.010
0.020
−1000
−800
−600
−400
−200
0
200
bp

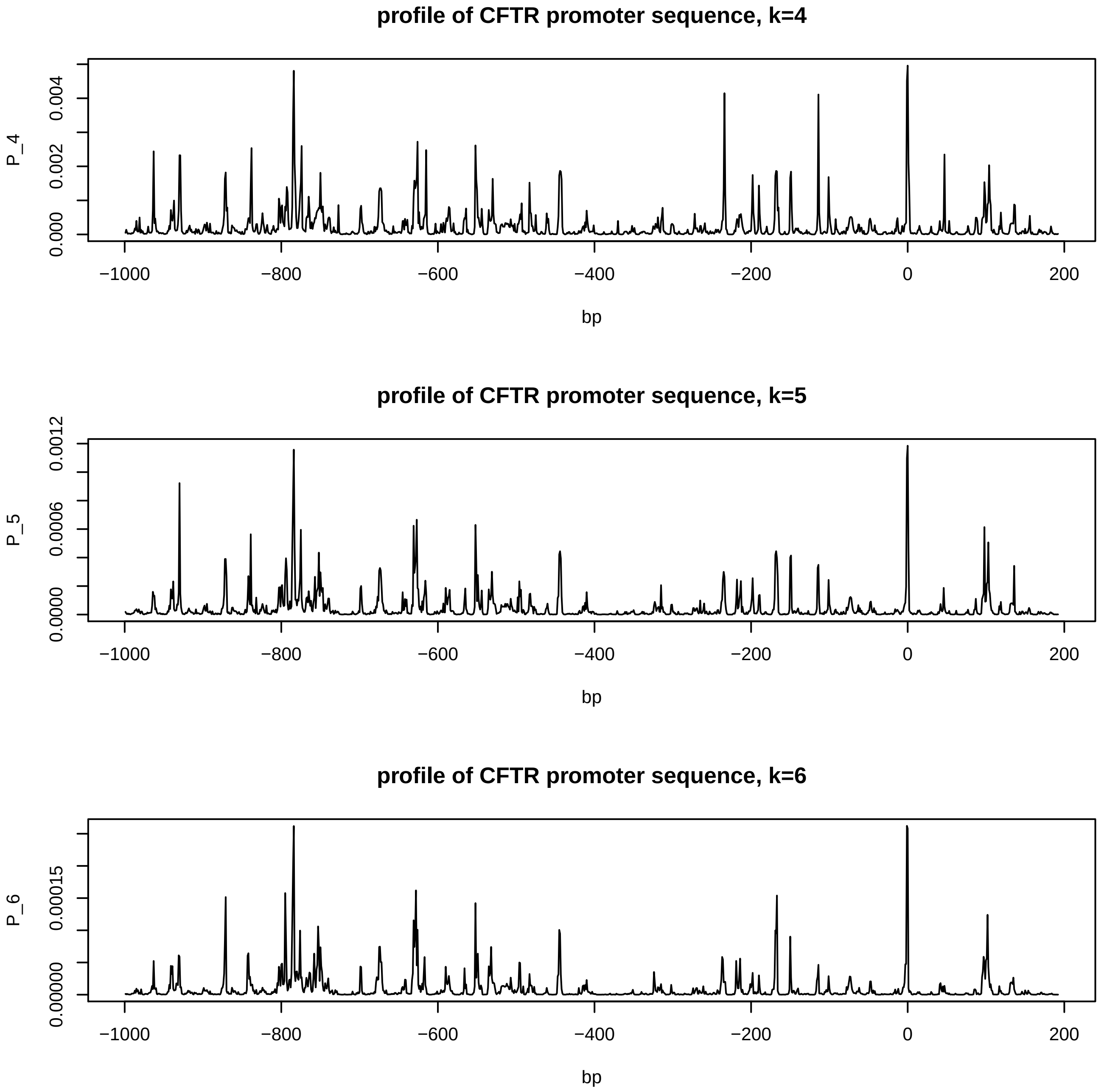
profile of CFTR promoter sequence, k=4
P_4
0.000
0.002
0.004
−1000
−800
−600
−400
−200
0
200
bp
profile of CFTR promoter sequence, k=5
P_5
0.0000
0.0006
0.0012
−1000
−800
−600
−400
−200
0
200
bp
profile of CFTR promoter sequence, k=6
P_6
0.00000
0.00015
−1000
−800
−600
−400
−200
0
200
bp

profile of CFTR promoter sequence, k=7

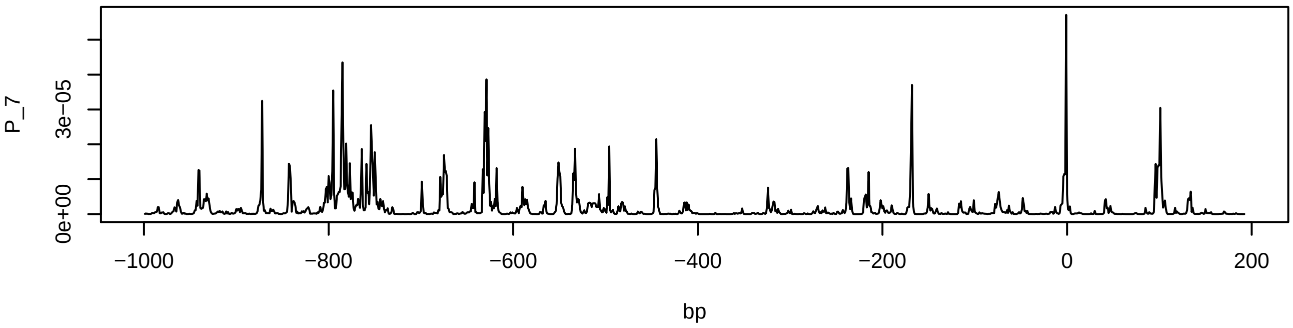

profile of CFTR promoter sequence, k=8

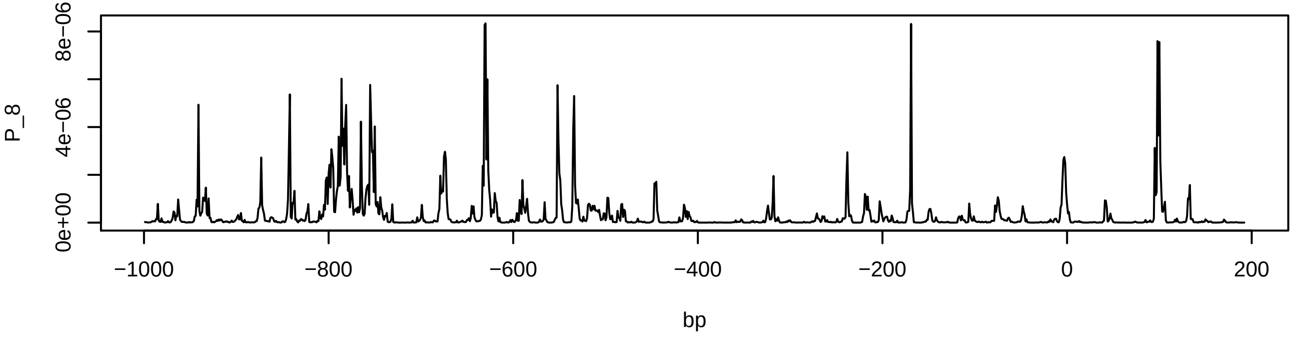

profile of CFTR promoter sequence, k=9

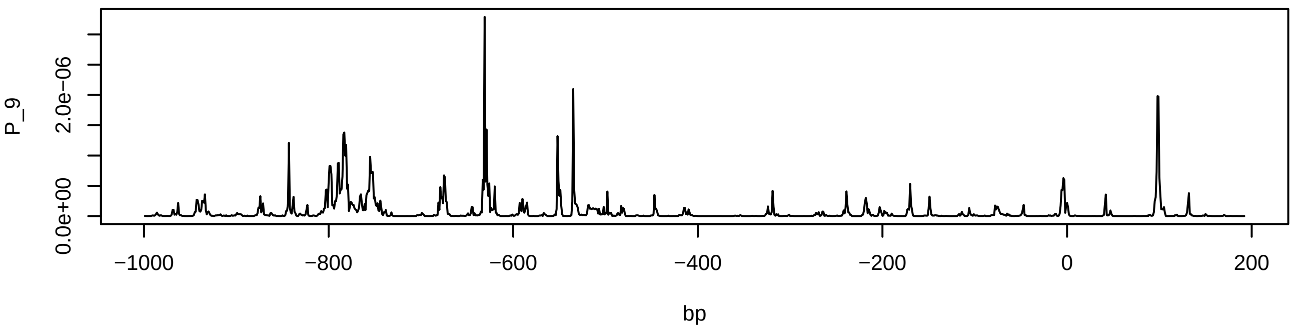

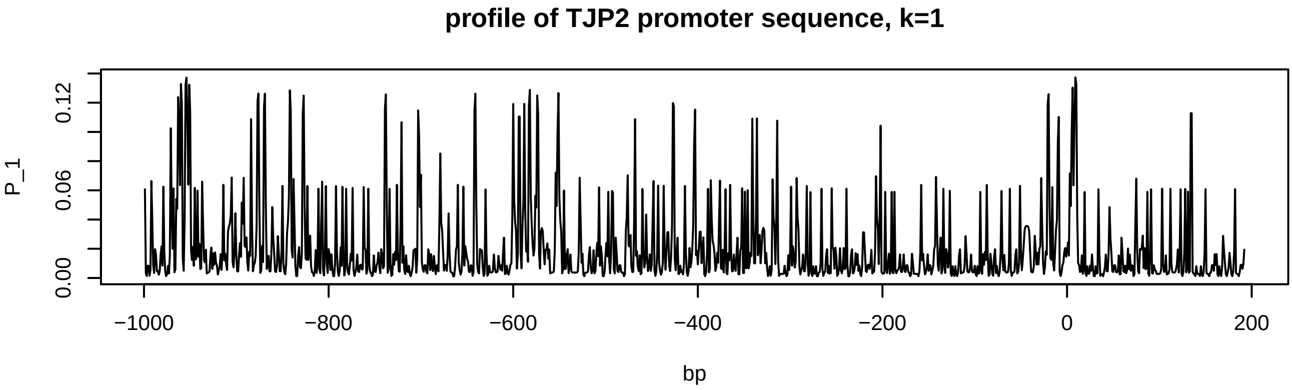

profile of TJP2 promoter sequence, k=1
P_1
0.00
0.06
0.12
−1000
−800
−600
−400
−200
0
200
bp

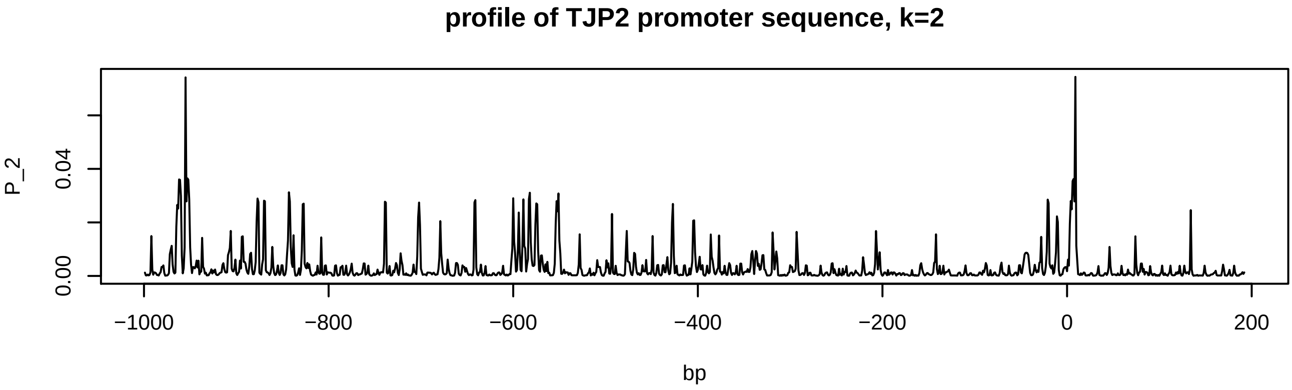

profile of TJP2 promoter sequence, k=2
P_2
0.00
0.04
−1000
−800
−600
−400
−200
0
200
bp

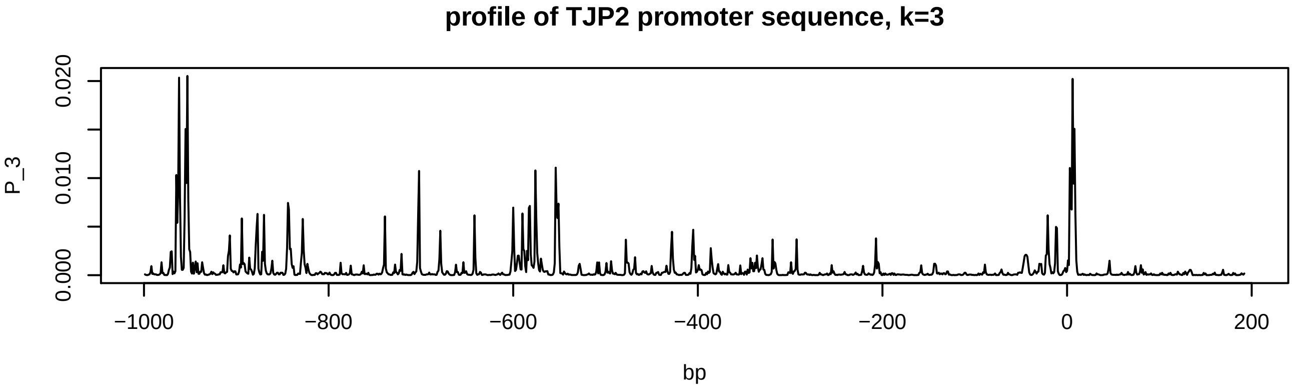

profile of TJP2 promoter sequence, k=3
P_3
0.000
0.010
0.020
−1000
−800
−600
−400
−200
0
200
bp

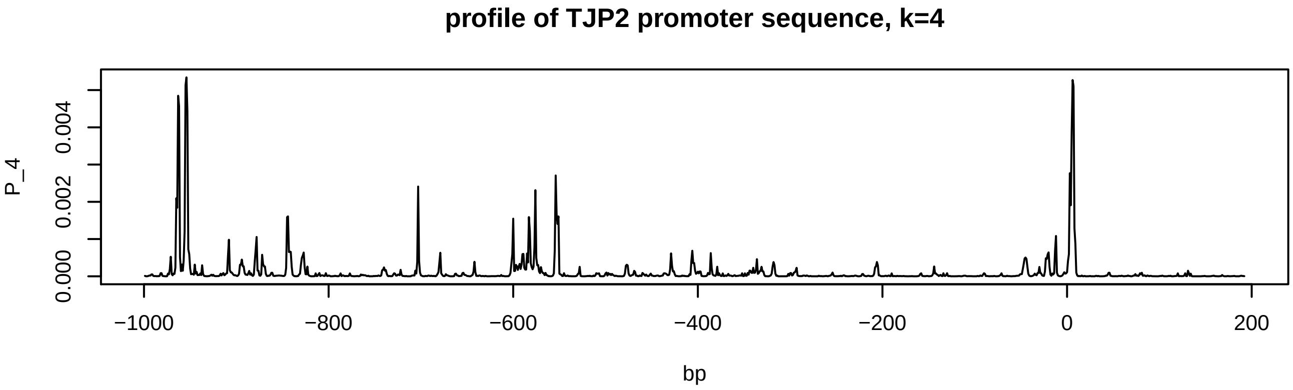
profile of TJP2 promoter sequence, k=4
P_4
0.000
0.002
0.004
−1000
−800
−600
−400
−200
0
200
bp

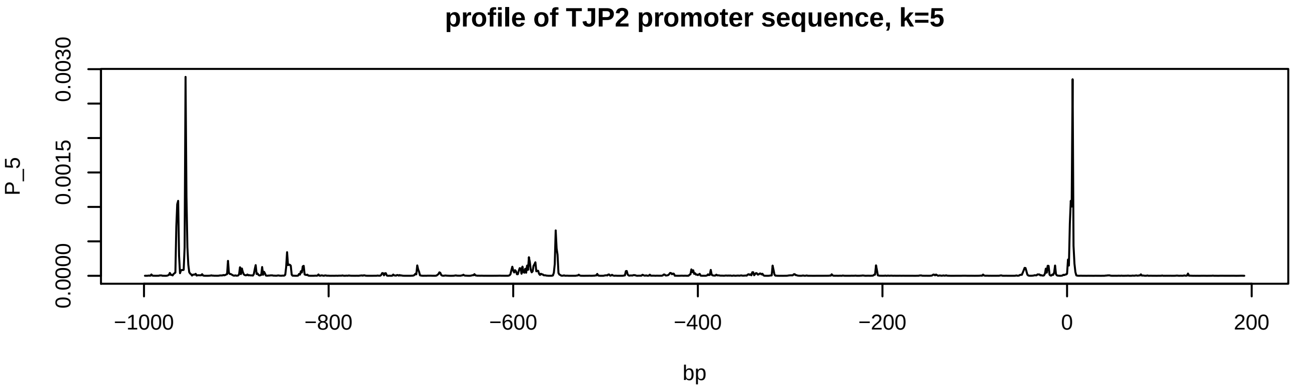
profile of TJP2 promoter sequence, k=5
P_5
0.0000
0.0015
0.0030
−1000
−800
−600
−400
−200
0
200
bp

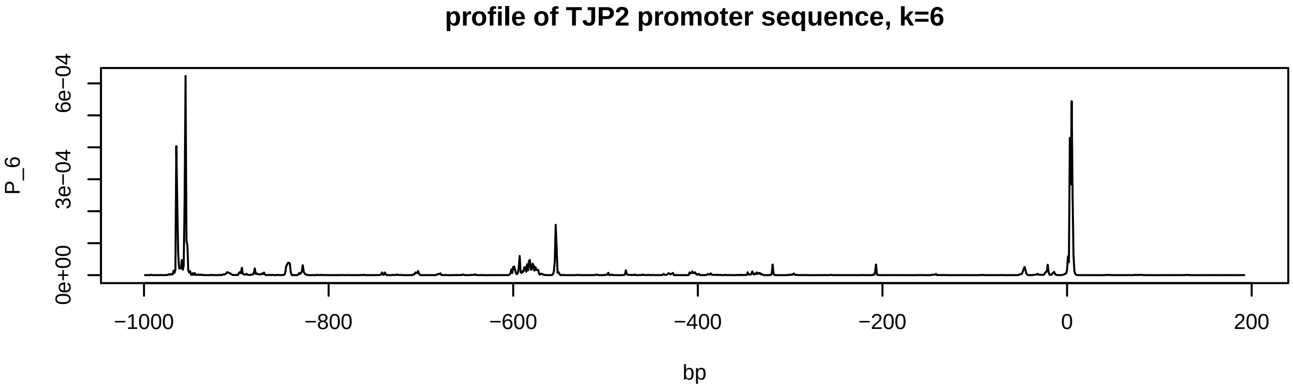
profile of TJP2 promoter sequence, k=6
P_6
0e+00
3e−04
6e−04
−1000
−800
−600
−400
−200
0
200
bp

profile of TJP2 promoter sequence, k=7

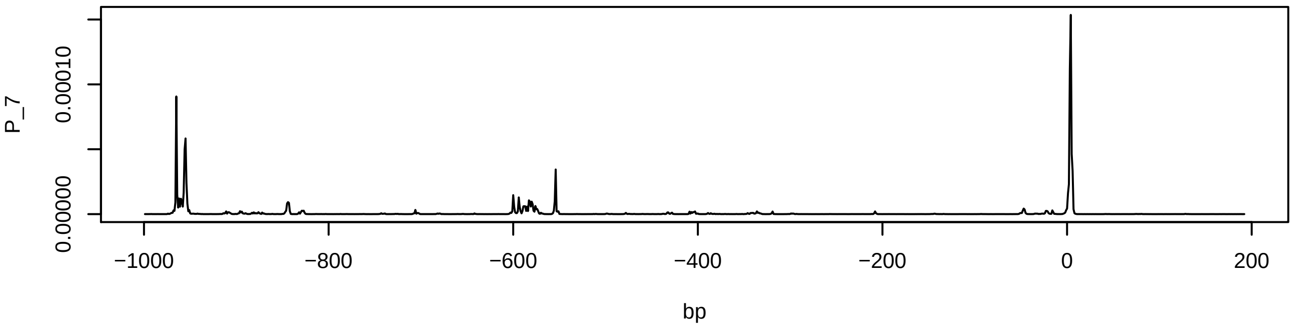

profile of TJP2 promoter sequence, k=8

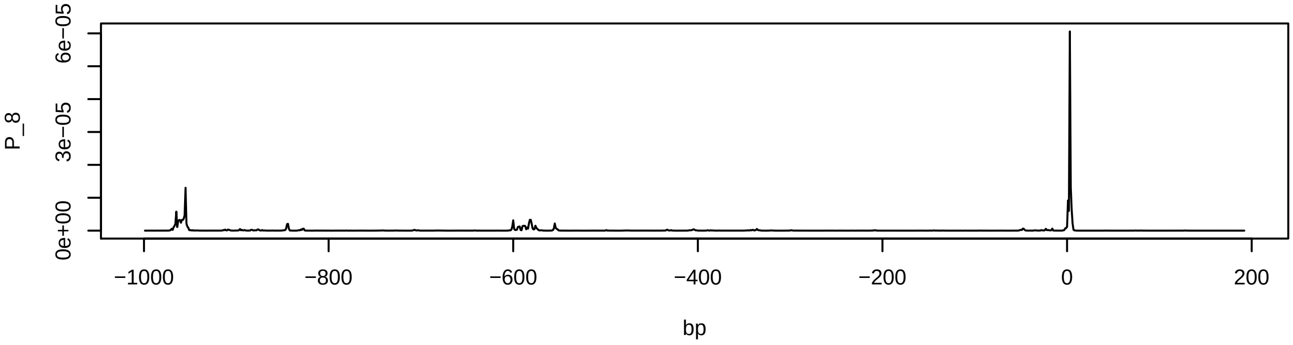

profile of TJP2 promoter sequence, k=9

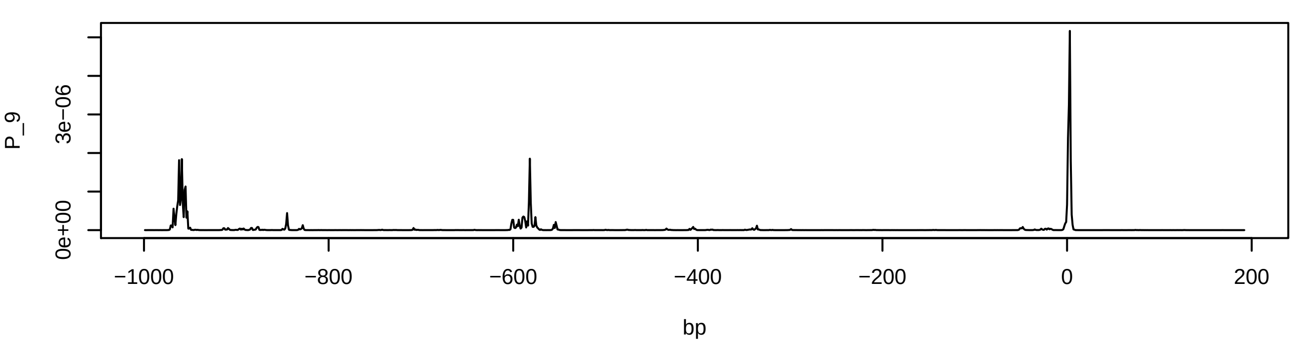

Supplement: Additional file 1 — Examples of DNA duplex instability profiles. This file contains figures showing the DNA duplex instability profiles for two promoter sequences, with bubble size ranging from k = 1 to k = 9 for each sequence. The genes used for these examples are CFTR and TJP2. [file 1471-2105-11-604-S1.PDF]
